# La experiencia europea ADVANTAGE para el manejo de la fragilidad: claves sobre su aplicabilidad en América Latina

**DOI:** 10.26633/RPSP.2021.107

**Published:** 2021-09-13

**Authors:** Cristina Alonso Bouzón, Leocadio Rodríguez Mañas

**Affiliations:** 1 Servicio de Geriatría, Hospital Universitario de Getafe GetafeMadrid España Servicio de Geriatría, Hospital Universitario de Getafe, Getafe, Madrid, España.

**Keywords:** Fragilidad, personas con discapacidad, envejecimiento saludable, dinámica poblacional, prevención de enfermedades, Frailty, disabled persons, healthy aging, population dynamics, disease prevention, Fragilidade, pessoas com deficiência, envelhecimento saudável, dinâmica populacional, prevenção de doenças

## Abstract

La Comisión Europea y 22 de sus Estados Miembros cofinanciaron durante 2017-2019 la primera acción conjunta para abordar la fragilidad en las personas mayores, denominada ADVANTAGE Joint Action. En el marco de esta iniciativa, se definió una estrategia común, basada en la mejor evidencia científica, para posicionar el envejecimiento saludable y la fragilidad como temas prioritarios de salud pública en los países participantes y contribuir así a un abordaje homogéneo de la fragilidad en toda Europa. En este artículo se detalla la metodología del trabajo realizado y los principales logros de ADVANTAGE y se incluye un análisis de las claves que contribuyeron a su éxito. En los tres años de funcionamiento se formaron y desarrollaron potentes redes de trabajo multisectoriales, tanto a nivel nacional como internacional. ADVANTAGE logró marcar rutas prácticas para abordar de manera global la fragilidad y la prevención de la dependencia en 22 países con realidades políticas, económicas, sociales y organizativas muy heterogéneas. ADVANTAGE ha demostrado que acciones de este tipo son factibles y eficaces, y que si se toman en cuenta y aplican los puntos clave de éxito identificados, debidamente ajustados a cada realidad, esta acción puede ser reproducible en cualquier país decidido a promover el envejecimiento saludable de su población, incluidos los de América Latina y el Caribe.

## Abstract (en)

The European Commission and 22 of its Member States cofinanced, in 2017-2019, the first joint action to address frailty in older persons, known as ADVANTAGE Joint Action. The initiative developed a common strategy, based on the best scientific evidence, to position healthy aging and frailty as priority public health issues in the participating countries and thus contribute to uniformly addressing frailty across Europe. This article details the methodology and main achievements of ADVANTAGE and includes an analysis of the key factors contributing to its success. In the Action’s three years, powerful multisectoral networks were formed and developed, both nationally and internationally. ADVANTAGE succeeded in mapping out practical routes for comprehensively addressing frailty and dependency prevention in 22 countries with very heterogeneous political, economic, social, and organizational realities. ADVANTAGE has demonstrated that actions of this type are feasible and effective, and that if the key points of success that have been identified are considered and applied, duly adjusted to each reality, this action can be replicable in any country resolved to promoting the healthy aging of its population, including those of Latin America and the Caribbean.

## RESUMO

De 2017 a 2019, a Comissão Europeia e 22 dos seus Estados Membros cofinanciaram a primeira ação conjunta para abordar a fragilidade em idosos, denominada ADVANTAGE Joint Action. No âmbito dessa iniciativa, foi definida uma estratégia comum, baseada nas melhores evidências científicas, para posicionar o envelhecimento saudável e a fragilidade como questões prioritárias de saúde pública nos países participantes, contribuindo, assim, para uma abordagem homogênea à fragilidade em toda a Europa. Este artigo detalha a metodologia do trabalho realizado e as principais conquistas da ADVANTAGE, e inclui uma análise das chaves que contribuíram para seu sucesso. Nos três anos de operação da iniciativa, foram formadas e desenvolvidas poderosas redes multissetoriais, tanto nacional quanto internacionalmente. A iniciativa ADVANTAGE foi capaz de criar roteiros práticos para abordar de maneira global a fragilidade e a prevenção da dependência em 22 países com realidades políticas, econômicas, sociais e organizacionais bastante heterogêneas. A ADVANTAGE mostrou que ações deste tipo são viáveis e eficazes, e que se os principais fatores de sucesso identificados forem levados em consideração e aplicados, devidamente ajustados a cada realidade, esta ação pode ser reproduzida em qualquer país determinado a promover o envelhecimento saudável de sua população, inclusive na América Latina e no Caribe.

El envejecimiento de la población es uno de los mayores retos que deberá afrontar la Humanidad en el siglo XXI (1). Se estima que el número de personas de 60 años o más crecerá de forma más acelerada en los países de renta baja y media: de 625 millones en 2017 a 1 700 millones en 2050, mientras que en los países de renta alta pasará de 310 millones a 427 millones (1). América Latina y el Caribe es la segunda región con más rápido crecimiento en el número de personas mayores, después de África (2), y se estima que para el 2050 la proporción de personas mayores de 60 años superará el 20% en todos los países, con un pico mayor en Costa Rica y Chile, donde se superará el 30% (1).

Las tendencias demográficas sugieren que aumentará el número de personas con discapacidad y dependencia asociadas al envejecimiento, lo que afectará no solo a la calidad de vida de esas personas sino también a la sostenibilidad de los sistemas sanitarios y sociales (3). Sin embargo, es posible actuar para evitar o reducir la progresión de las personas mayores hacia la discapacidad y la dependencia, lo que permitiría prolongar la vida libre de discapacidad, con mayor independencia y calidad (4). Preocupa también el aumento previsto de los costos de la salud y la atención a largo plazo, además de las consecuencias económicas que traerá la disminución proporcional del número de personas en edad de trabajar (2). No obstante, atender a la población de personas mayores no tiene por qué tener necesariamente un costo elevado y podría mostrar una buena relación de costo-efectividad, ya que estas personas pueden generar beneficios económicos y sociales, especialmente si se mantienen saludables y activas (2).

La Organización Mundial de la Salud (OMS) define el envejecimiento saludable como el proceso de desarrollar y mantener una capacidad funcional que permita el bienestar de las personas mayores (1). La OMS defiende que una de las claves para fomentar el envejecimiento saludable es prevenir la dependencia, lo que se puede alcanzar si se logran identificar y abordar oportunamente las condiciones asociadas con la disminución de la capacidad funcional (1). Por lo tanto, identificar a tiempo las condiciones que preceden a la discapacidad y la dependencia es un requisito esencial para promover eficazmente el envejecimiento saludable (5).

## LA FRAGILIDAD COMO DIANA PARA FAVORECER EL ENVEJECIMIENTO SALUDABLE DE LA POBLACIÓN

La fragilidad es la condición más importante del deterioro funcional asociado al envejecimiento (5) y se caracteriza por el declinar progresivo de los sistemas fisiológicos relacionado con el envejecimiento, que resulta en una reducción de todas las capacidades físicas y mentales de la persona mayor. Esto confiere una extrema vulnerabilidad a estresores, lo que aumenta el riesgo de aparición de diversos eventos adversos para la salud (6). Se ha comprobado que la prevención y el manejo precoz de la fragilidad pueden evitar muchos de los eventos adversos asociados con el envejecimiento no saludable, incluidas la discapacidad y la dependencia (1).

Sin embargo, aunque se sabe que abordar la fragilidad es un paso necesario para promover el envejecimiento saludable y es —sin duda— uno de los mayores retos de la salud pública mundial (7-9), en la mayoría de los países no se considera la fragilidad una prioridad de salud.

La preocupación por esta problemática llevó a la Comisión Europea a incluir en el Tercer Programa Europeo de Salud 2014-2020 la primera acción conjunta para abordar integralmente la fragilidad en las personas mayores, denominada ADVANTAGE Joint Action —en adelante ADVANTAGE—, centrada en abordar la fragilidad para la promoción del envejecimiento saludable en Europa.

En este artículo se detalla cómo ADVANTAGE ha desarrollado su trabajo y cuáles han sido los principales logros, y se analizan las claves que contribuyeron a su éxito y que se deben tener en cuenta para extender esta iniciativa a otros entornos.

## EUROPA PLANTA CARA A LA FRAGILIDAD: LA ACCIÓN CONJUNTA ADVANTAGE

Las acciones conjuntas son proyectos de la Unión Europea para desarrollar acciones cofinanciadas por sus Estados Miembros —en las que en ocasiones pueden participar también países que no son miembros— con el objetivo de avanzar en temas considerados clave para mejorar la salud de los ciudadanos europeos.

El objetivo de ADVANTAGE, como acción conjunta, era definir una estrategia común que posicionara el envejecimiento saludable y la fragilidad como temas prioritarios de salud pública en los Estados Miembros, y contribuir a impulsar el abordaje de la fragilidad, según la mejor evidencia científica, de una manera más homogénea en todos ellos. En consecuencia, se mejoraría la prevención, la detección, la evaluación y el manejo de la fragilidad, y con ello se promovería el envejecimiento saludable. Esta iniciativa se convirtió en un excelente ejemplo de buena práctica en salud pública.

Con una duración de tres años (2017-2019), en ADVANTAGE participaron 38 organizaciones europeas procedentes de 22 de los 26 Estados Miembros de aquel entonces. Los países que formaron parte de la acción conjunta son Alemania, Austria, Bélgica, Bulgaria, Croacia, Chipre, Eslovenia, Finlandia, Francia, Grecia, Holanda, Hungría, Irlanda, Italia, Lituania, Malta, Noruega, Polonia, Portugal, Reino Unido y Rumanía, además de España que fungió como país coordinador, a través del Hospital Universitario de Getafe del Servicio Madrileño de Salud.

Para alcanzar su objetivo, ADVANTAGE se propuso las siguientes líneas de acción:

1. Revisión de la bibliografía científica existente sobre el abordaje de la fragilidad, tanto a nivel individual como poblacional, con el fin de identificar el modelo ideal —o *gold stantard—* de abordaje integral de la fragilidad para aplicar en Europa. Estas revisiones sistemáticas se realizaron sin excluir ningún idioma y sin fechas límites, y abarcaron también la llamada literatura gris; la metodología se explica con mayor detalle en las publicaciones resultantes (6, 10-20)
2. Recopilación de información sobre el estado del abordaje de la fragilidad al inicio del proyecto en los 22 Estados Miembros participantes. Era importante conocer las principales acciones que se estaban llevando a cabo en cada país, identificar los actores involucrados, las circunstancias nacionales y las posibles barreras que entorpecían la implementación de las acciones.
3. Desarrollo de un marco teórico común para diseñar políticas efectivas de prevención y abordaje de la fragilidad en Europa, tomando en cuenta las líneas de acción previas, en particular el estado de la situación de la que se partía y a qué se aspiraba en cada país participante.
4. Proposición de hojas de ruta específicas para cada Estado Miembro a partir del marco teórico común, con metas a corto, mediano y largo plazos. Para ello fue necesario involucrar desde el inicio al mayor número de actores nacionales posibles (políticos, profesionales sanitarios y no sanitarios, personas mayores y cuidadores, entre otros). Estas hojas de ruta estarían adaptadas a cada contexto específico, teniendo en cuenta no solo las necesidades y las prioridades nacionales sino también la etapa de desarrollo e implementación de las políticas de envejecimiento y sus circunstancias sociales, sanitarias, económicas y políticas. Las autoridades nacionales encargadas de las políticas sanitarias y sociales debían participar siempre que fuera posible, con el fin de que estas hojas de ruta se aprobaran a nivel ministerial y se adoptara un compromiso de acción real para los próximos años.
5. Identificación de lagunas en el conocimiento sobre el envejecimiento —especialmente sobre la fragilidad— entre los responsables de formular políticas y sus gestores, los profesionales sanitarios y no sanitarios, y las personas mayores y sus cuidadores. Esta línea de acción incluía el reconocimiento y la creación de conciencia sobre la necesidad de entrenar y formar a todos esos actores, especialmente a las personas mayores y sus cuidadores (formales y no formales), con el objetivo de promover su empoderamiento y facilitar un mayor apoyo al autocuidado de las personas mayores.
6. Consulta con expertos y reconocidos actores nacionales e internacionales no involucrados en ADVANTAGE (entidades afiliadas, panel de académicos expertos y comité asesor externo) con el objetivo de lograr su apoyo y enriquecer el trabajo realizado a lo largo de estos años. Esto agregaría valor a las discusiones, dado el conocimiento y experiencia de estos actores en temas de envejecimiento, salud pública, fragilidad y enfermedades crónicas.
7. Difusión del enfoque común en los países participantes, creación de conciencia —no solo en los actores involucrados en el envejecimiento, reales y potenciales, sino también en la población general— sobre qué es la fragilidad, la necesidad de abordarla y cómo hacerlo para reducir la discapacidad y la dependencia, y disminuir la demanda futura de cuidados a largo plazo.

Para llevar a cabo estas líneas de acción se establecieron grupos de trabajo que operaban a dos niveles complementarios: mientras la actividad regional de cada grupo de trabajo la ejecutaban representantes de varios países, la actividad nacional —encargada de la recogida de la información, la planificación y la difusión de los acuerdos en los países— la coordinaban representantes de cada Estado Miembro participante, designados por los respectivos ministerios de salud o de servicios sociales. Las actividades consistían básicamente en hacer revisiones de la bibliografía especializada —incluidos la literatura gris y los documentos de uso interno (en los ámbitos local, provincial, nacional y regional)—, organizar reuniones y foros de discusión, conceptualizar y articular ideas, y consultar a expertos.

El trabajo se estructuró de la siguiente manera:

- En el primer año (2017) se preparó el Informe Sobre el Estado del Arte en Prevención y Manejo de la Fragilidad (6), a partir de la revisión de la información científica disponible sobre el tema; este informe concluye con mensajes clave que representan el modelo ideal al que deben aspirar los Estados Miembros al abordar la fragilidad.
- En el segundo año (2018), los representantes de los Estados Miembros participantes respondieron un cuestionario; sus respuestas permitieron conocer cómo se estaba abordando la fragilidad en cada país en ese momento y la concordancia de esas acciones con las recomendaciones del informe elaborado el año anterior (6).
- En el último año de ADVANTAGE (2019), a partir del modelo ideal al que se debe aspirar y el trabajo desarrollado en cada Estado Miembro participante, los grupos multisectoriales nacionales trabajaron en la elaboración de hojas de ruta para avanzar en el abordaje de la fragilidad. Si bien cada país elaboró su propia hoja de ruta, se celebraron reuniones de todos los Estados Miembros (en Atenas y Hannover) para identificar no solo las actividades comunes, sino también los requerimientos mínimos de esas actividades y sus indicadores.

Las actividades para crear conciencia sobre el problema fueron transversales y se realizaron a lo largo de los tres años de duración de la acción conjunta.

### Los resultados de ADVANTAGE

Las actividades realizadas en el marco de ADVANTAGE han generado resultados tangibles, en particular numerosos documentos, publicaciones y foros de discusión (cuadro 1). Todos estos resultados se han reunido en un documento central titulado Promocionando el Envejecimiento Saludable a Través de un Abordaje Preventivo de la Fragilidad (FPA, por las siglas en inglés de *Frailty Prevention Approach*) (21). Además de las principales conclusiones de las revisiones bibliográficas realizadas, este documento incluye recomendaciones claras y concretas para abordar la prevención, la detección y el tratamiento de la fragilidad de una manera efectiva y homogénea en toda Europa. Estas recomendaciones surgen del acuerdo entre los 22 Estados Miembros participantes y las 38 organizaciones que colaboraron en este proyecto.

Por lo tanto, el FPA está llamado a ser el documento guía para el fortalecimiento de las capacidades nacionales en Europa y debe contribuir a acelerar el desarrollo de políticas nacionales y locales que apoyen el envejecimiento saludable, centradas en la prevención y el abordaje integral de la fragilidad. El FPA brinda orientación a los actores involucrados en el desarrollo de políticas nacionales y locales para abordar el envejecimiento, como son los encargados directos de tomar decisiones políticas, los asesores técnicos, los gerentes, los profesionales de la salud, los cuidadores y los académicos, entre otros.

El FPA puede ser una valiosa herramienta para:

- Identificar áreas de acción relacionadas con la fragilidad, tanto a nivel nacional como local, y posicionarlas entre las prioridades de salud
- Desarrollar objetivos nacionales y locales para cada área de acción y definir los resultados esperables
- Implementar intervenciones probadas para reducir los factores de riesgo de trastornos que llevan a la fragilidad
- Implementar intervenciones probadas para abordar la fragilidad, tanto a nivel individual como poblacional
- Implementar intervenciones probadas para formar a los trabajadores, especialmente, pero no solo, en el área de la salud
- Medir el progreso y los resultados según los indicadores propuestos
- Llevar la acción más allá del sector de la salud mediante actividades de cooperación multisectorial
- Promover la colaboración local e internacional para transferir y escalar el uso de las buenas prácticas que abordan la fragilidad

Las recomendaciones del FPA se estructuraron en 10 dominios o áreas de trabajo que incluían todas las actividades que deben llevar a cabo las autoridades involucradas en el manejo de la fragilidad (cuadro 2), además de la justificación teórica, los posibles indicadores de resultado y ejemplos de buenas prácticas que se deben seguir.

Sin embargo, los resultados de ADVANTAGE van más allá de los documentos generados: durante los tres años de funcionamiento se han formado y desarrollado potentes redes de trabajo multisectoriales, tanto a nivel nacional como internacional.

A nivel nacional, la mayoría de los grupos y redes se mantienen activos aún al redactar esta nota (junio de 2021). Estos grupos fueron los encargados de involucrar progresivamente durante el proyecto a los representantes de todos los sectores relacionados con el envejecimiento —incluidos las autoridades nacionales de salud—, desarrollar el mapa de ruta para los próximos años y lograr el compromiso de todos los actores de implementarlo. Al término de la acción conjunta, en 2019, nueve de los Estados Miembros participantes habían incorporado los resultados de ADVANTAGE en sus planes —nacionales o locales— y documentos políticos: Austria, Bulgaria, España, Finlandia, Irlanda, Italia, Malta, Rumania y el Reino Unido. A estos resultados contribuyó el hecho de que desde la formación de estos grupos se trabajó para su permanencia más allá de la duración de ADVANTAGE, a fin de garantizar la implementación de las medidas en los Estados Miembros y la evaluación del cumplimiento de sus compromisos. Un ejemplo de los avances logrados es la constitución de un grupo de trabajo de fragilidad, permanente y activo, en el Ministerio de Sanidad de España.

**CUADRO 1.** Resultados de la iniciativa Acción Conjunta ADVANTAGE según sus líneas de acción, 2017-2019

Línea de acción	Resultados
1.Revisión de la bibliografía científica existente sobre el abordaje de la fragilidad	Se publicó un artículo con estos resultados (6) y se elaboraron revisiones de actualización del marco común (no publicadas)
2. Recopilación de información sobre el estado del abordaje de la fragilidad en los 22 Estados Miembros participantes al inicio del proyecto	Informe de cada Estado Miembro sobre las áreas específicas de prevención, detección temprana, manejo, sistema integrado de salud y formación e investigación en este tema
3. Desarrollo de un marco teórico común para diseñar políticas efectivas de prevención y abordaje de la fragilidad en Europa	Estrategia global Promocionando el Envejecimiento Saludable a Través de un Abordaje Preventivo de la Fragilidad (21)
4. Proposición de hojas de ruta específicas para cada Estado Miembro	Mapa de ruta de cada Estado Miembro
5. Identificación de lagunas en el conocimiento sobre el envejecimiento en los distintos actores	Cuadro de capacidades mínimas multiprofesionales para prevenir y abordar la fragilidad (13)
6. Consulta con expertos y reconocidos actores nacionales e internacionales no involucrados directamente en la iniciativa	Múltiples reuniones, debates, discusiones, talleres de trabajo y otros foros de consulta
7. Difusión del enfoque común en los países participantes	Más de 20 artículos en revistas científicas (6, 10-20)^a^, 85 artículos en medios de comunicación masiva, 49 reuniones (técnicas, científicas y divulgativas), y 146 ponencias y 71 comunicaciones en congresos, entre otras

***Fuente:*** elaborado por los autores.

a Se consideran solamente los artículos publicados en revistas científicas de la llamada corriente principal.

**CUADRO 2.** Actividades recomendadas por la iniciativa Acción Conjunta ADVANTAGE para la implementación del abordaje de la fragilidad, 2017-2019

Dominio	Actividades
1. Concienciación de la población, involucramiento de los socios y empoderamiento de las personas mayores	- Campañas de concienciación - Involucramiento de los socios clave
2. Compromiso con la iniciativa para el abordaje de la fragilidad	- Desarrollo de una estrategia nacional sobre el envejecimiento saludable y la fragilidad - Alineamiento de otras estrategias previas - Creación de un departamento o programa especializado en los misterios de salud
3. Promoción del envejecimiento saludable y prevención de la fragilidad	- Implementación de estrategias poblacionales - Promoción de las ciudades amigables con la edad - Elaboración de guías para la promoción del envejecimiento saludable
4. Detección precoz de la fragilidad	- Desarrollo de iniciativas para la detección temprana de la fragilidad - Inclusión de la fragilidad en las encuestas nacionales de salud - Adopción de estrategias de estratificación del riesgo de la población a partir de datos epidemiológicos
5. Manejo adecuado de la fragilidad	- Utilización de la valoración geriátrica integral, adaptada a cada ámbito de atención - Desarrollo de guías para el manejo de la fragilidad e intervenciones específicas
6. Establecimiento y mejoramiento continuado de un modelo integrado de cuidado para abordar la fragilidad	- Desarrollo de recomendaciones para mejorar el modelo de cuidados integrados - Desarrollo de un programa que asegure los cuidados intermedios y el manejo de las transiciones - Evaluación y mejora de los servicios, replica de programas con resultados positivos y pilotaje de nuevos programas
7. Educación y entrenamiento	- Inclusión en el currículo de todas las disciplinas sanitarias y sociales de las recomendaciones sobre las capacidades mínimas en pregrado, postgrado y formación continuada, contenidas en el documento Promocionando el Envejecimiento Saludable a Través de un Abordaje Preventivo de la Fragilidad (21)
8. Investigación	- Apoyo en la creación de redes de investigación multidisciplinarias - Promoción de la cooperación con grupos internacionales - Aseguramiento de convocatorias de investigación sobre fragilidad
9. Apoyo a la implementación (financiación y equipamiento)	- Asignación de recursos para la implementación de las estrategias nacionales para el manejo del envejecimiento y la fragilidad - Desarrollo de plataformas de información compartida para facilitar los cuidados integrados, el abordaje de la fragilidad y la educación continuada
10. Monitoreo de la calidad y evaluación de la relación costo-efectividad de las intervenciones	- Inclusión de indicadores de fragilidad en los objetivos de salud - Utilización de indicadores cualitativos - Evaluación y mejora continua de los servicios de salud y sanidad

***Fuente:*** elaborado por los autores.

A nivel internacional, los miembros de ADVANTAGE colaboraron entre sí y con diversos organismos e instituciones, como la Comisión Europea, la Sociedad Europea de Medicina Geriátrica, la Fundación Internacional de Cuidados Integrados y gran parte de las sociedades nacionales de geriatría. Estas colaboraciones dieron lugar a numerosas presentaciones en eventos y publicaciones (10-19), que han generado un fluido intercambio, entre otras cosas, de buenas prácticas de abordaje de la fragilidad. Al día de hoy, gran parte de los miembros de ADVANTAGE siguen colaborando en el grupo de fragilidad de la Fundación Internacional de Cuidados Integrados.

Los miembros de ADVANTAGE, junto a las entidades afiliadas al proyecto y los académicos europeos consultados, confían en que la adopción y aplicación de las recomendaciones incluidas en el FPA contribuirán a reducir la prevalencia de la discapacidad y la dependencia asociadas al envejecimiento en Europa. Aunque la pandemia de COVID-19 ha frenado una gran parte de las actividades que se habían generado en el marco de ADVANTAGE, por el énfasis puesto en ella, se espera que puedan retomarse una vez superada esta crisis sanitaria.

## CLAVES PARA EXTENDER LA INICIATIVA ADVANTAGE A OTRAS REGIONES

La acción conjunta ADVANTAGE constituye una referencia para cualquier región que se proponga responder a los retos del envejecimiento y la fragilidad, incluida América Latina y el Caribe. Si a pesar de las enormes diferencias existentes entre los sistemas de salud, las políticas sociales y sanitarias, y los contextos económicos, culturales y sociales de los 22 Países Miembros de la Unión Europea que participaron en ADVANTAGE, se lograron estos resultados, podría afirmarse que cualquier región o país podría desarrollar una iniciativa similar.

Aunque centrada en los aspectos de organización y funcionamiento de los sistemas de salud europeos y con un explícito y decidido interés en ajustar las acciones a las condiciones particulares de cada país participante, ADVANTAGE ha validado un modelo de evaluación de la situación, sistematización de la información y transformación de esos insumos en directrices concretas de actuación. Por haberse concebido para ser adaptada a las condiciones de cada país particular, es aplicable en otros entornos y realidades sociales y sanitarias.

Algunas de las claves que contribuyeron al éxito de esta iniciativa y que, por lo tanto, se deben tener en cuenta para extrapolarla son:

- Incluir a todos los países de la región, independientemente del nivel de implementación de políticas relacionadas con el envejecimiento y la fragilidad. En la acción conjunta europea, tal y como se puede ver en el FPA (21), los resultados de la evaluación inicial muestran realidades muy heterogéneas en varias áreas o dominios de la implementación de las políticas. Aunque hubo países que mostraron un nivel de implementación avanzado y sostenible —lo que demostró que adquirir un alto nivel de implementación era posible—, gran parte de los Estados Miembros se encontraban en un estado de implementación básico en algunas o todas las áreas o dominios^2^. El intercambio de buenas prácticas entre los países con mayor y menor niveles de implementación, y las discusiones en las que participaron todos los Estados Miembros sobre las barreras, los éxitos y las estrategias que desarrollaron o evitaron fue lo que más ayudó a establecer recomendaciones que se pudieran llevar a cabo en todos los niveles de implementación y, por lo tanto, aplicables en cualquier Estado Miembro participante.
- Crear grupos multisectoriales con agentes locales desde el inicio. Si bien el grueso del trabajo de los grupos nacionales multisectoriales estaba programado para el tercer año de ejecución, los Estados Miembros que lograron implicar a todos o gran parte de los actores trabajaron en la formación de los grupos de una manera progresiva desde el inicio. Esta fue una de las claves del éxito de ADVANTAGE, ya que facilitó localizar los materiales bibliográficos locales o no publicados, y hacer un análisis detallado y profundo de cada realidad. Esto permitió, además, ajustar el marco teórico a la heterogeneidad existente en Europa y adaptar cada mapa de ruta a las circunstancias específicas de cada país. Se constató que los Estados Miembros que alcanzaron un mayor nivel de implementación fueron los que lograron involucrar a las personas mayores —y empoderarlas— y a los tomadores de decisiones —e imbuirlos de un compromiso real—, e incluir las recomendaciones en documentos políticos. Entre los agentes locales participantes en los grupos multisectoriales no deben faltar los profesionales sanitarios y no sanitarios, los cuidadores no formales y, por supuesto, la academia.
- Dedicar tiempo a unificar la visión y la misión del grupo multisectorial. El envejecimiento es un área del conocimiento típicamente multifacética en la que concurren diferentes visiones. A esto contribuye el hecho de que el concepto de fragilidad se utiliza de muy disímiles maneras, lo que da lugar a una polisemia que es fuente de confusión. No unificar debidamente la visión del grupo en algunos Estados Miembros fue una de las causas principales de no conseguir el compromiso de todos los agentes en esos países.
- Identificar desde el inicio al coordinador principal y los líderes de los grupos de trabajo. En una acción conjunta en la que participaron 22 países, 38 organizaciones y más de 100 personas surgieron numerosas dificultades y barreras no previstas. Además, es necesario tener claras las metas, los resultados esperados, la distribución de los recursos y la planificación de las etapas y plazos si se quiere garantizar el cumplimiento de los objetivos pactados.
- Disponer de recursos. La inversión económica es indispensable para poder implementar acciones como ADVANTAGE.

En conclusión, ADVANTAGE mostró ser una vía factible y productiva para posicionar el envejecimiento saludable y la fragilidad entre las prioridades de salud pública en los países participantes. Además, marcó rutas prácticas para la puesta en marcha y el monitoreo de programas basados en resultados científicos comprobados para abordar de manera global el tema de la fragilidad y la prevención de la dependencia. En ADVANTAGE participaron 22 Estados Miembros de la Unión Europea que tienen realidades sociales y sanitarias muy diversas; sin embargo, esto no impidió que esas rutas prácticas se adaptaran a las diferentes realidades económicas, políticas, sociales y organizativas de cada uno de los países a partir de un marco teórico común desarrollado y adoptado por todos. Se puede afirmar que, si se toman en cuenta y aplican debidamente los puntos clave de éxito identificados y se ajustan apropiadamente, la iniciativa ADVANTAGE es reproducible en prácticamente cualquier país decidido a promover el envejecimiento saludable de su población, incluidos los de América Latina y el Caribe.

## Declaración de los autores.

Las opiniones expresadas en este artículo son responsabilidad de los autores y no reflejan necesariamente los criterios ni la política de la *Revista Panamericana de Salud Pública / Pan American Journal of Public Health* y/o de la Organización Panamericana de la Salud.

## Footnotes

2 El nivel de implementación se clasificó como básico, débil, bien desarrollado, o avanzado y sostenible.
